# Multi-fractal modeling of curcumin release mechanism from polymeric nanomicelles

**DOI:** 10.1080/10717544.2022.2118402

**Published:** 2022-09-05

**Authors:** Camelia E. Iurciuc (Tincu), Marcel Popa, Leonard I. Atanase, Ovidiu Popa, Lacramioara Ochiuz, Paraschiva Postolache, Vlad Ghizdovat, Stefan A. Irimiciuc, Maricel Agop, Constantin Volovat, Simona Volovat

**Affiliations:** a Department of Pharmaceutical Technology, Faculty of Pharmacy, “Grigore T. Popa” University of Medicine and Pharmacy, Iaşi, Romania; b Department of Natural and Synthetic Polymers, Faculty of Chemical Engineering and Protection of the Environment, Gheorghe Asachi” Technical University, Iaşi, Romania; c Academy of Romanian Scientists, Bucharest, Romania; d Faculty of Dental Medicine, “Apollonia” University of Iasi, Iași, Romania; e Department of Emergency Medicine, “Grigore T. Popa” University of Medicine and Pharmacy, Iasi, Romania; f Department of Pharmaceutical and Biotechnological Drug Industry, “Grigore T. Popa” University of Medicine and Pharmacy, Iasi, Romania; g 1^st^ Medical Department, “Grigore T. Popa” University of Medicine and Pharmacy, Iasi, Romania; h Department of Biophysics and Medical Physics, “Grigore T. Popa” University of Medicine and Pharmacy Iasi, Iasi, Romania; i National Institute for Laser, Plasma and Radiation Physics, Bucharest, Romania; j Department of Physics, “Gh. Asachi” Technical University of Iasi, Iasi, Romania; k Department of Medical Oncology Radiotherapy, “Grigore T. Popa” University of Medicine and Pharmacy Iasi, Iasi, Romania; l Center of Oncology Euroclinic, Iasi, Romania

**Keywords:** amphiphilic copolymers, micelles, drug release kinetics, fractal model

## Abstract

The physicochemical properties of “smart” or stimuli-sensitive amphiphilic copolymers can be modeled as a function of their environment. In special, pH-sensitive copolymers have practical applications in the biomedical field as drug delivery systems. Interactions between the structural units of any polymer-drug system imply mutual constraints at various scale resolutions and the nonlinearity is accepted as one of the most fundamental properties. The release kinetics, as a function of pH, of a model active principle, i.e., Curcumin, from nanomicelles obtained from amphiphilic pH-sensitive poly(2-vinylpyridine)-b-poly(ethylene oxide) (P2VP-b-PEO) tailor-made diblock copolymers was firstly studied by using the Rietger-Peppas equation. The value of the exponential coefficient, n, is around 0.5, generally suggesting a diffusion process, slightly disturbed in some cases. Moreover, the evaluation of the polymer-drug system’s nonstationary dynamics was caried out through harmonic mapping from the usual space to the hyperbolic one. The kinetic model we developed, based on fractal theory, fits very well with the experimental data obtained for the release of Curcumin from the amphiphilic copolymer micelles in which it was encapsulated. This model is a variant of the classical kinetic models based on the formal kinetics of the process.

## 1. Introduction

Amphiphilic copolymers of suitable composition can self-assemble in an aqueous or organic medium in the form of objects, typically micelles, with sizes in the nanometric range. These micelles consist of a hydrophobic core and a hydrophilic corona (Riess, 2003; Atanase et al., 2014; Atanase et al., 2017). Moreover, in the case of drug-loaded micelles, the corona stabilizes the micelle and improves its biocompatibility, while ensuring the protection of the loaded active principle. Due to the stealth provided by the corona, the micelles are not destroyed, being invisible to the immune system, and thus the circulation time in the blood is prolonged (Iurciuc-Tincu et al., 2020).

A new class of materials is attracting a lot of interest, the so-called “smart” or stimuli-sensitive polymers whose physicochemical properties can be adapted in response to a change in their environment. This change, which may be of a physical or chemical nature, is most often reversible; it results in a rapid modification of the polymer microstructure (shape, surface characteristics, solubility) by the action of a stimulus (Adibfar et al., 2020).

In particular pH- and thermo-sensitive copolymers, for which the external conditions are relatively easy to modify, can be used to create stimuli-sensitive micellar systems (Atanase & Riess, 2013; Atanase et al., 2017; Winninger et al., 2019). The variation of pH affects ionic interactions, hydrogen bonds and hydrophobic interactions and therefore modifies the hydrophilic/hydrophobic balance, which affects the solubility of the polymer in an aqueous medium (the polymer chains are contracted or extended). A change in the environment pH of a polymer containing ionizable groups causes a change in the degree of ionization to a specific value called pKa.

These pH-sensitive systems are attracting a wide interest, especially in the biomedical field due to the different pH values encountered in blood, organs, cells. Thus, the variation of pH can be used to generate disaggregation of these nanocarriers into unimers or even to modify their morphology and subsequently to control the release of the loaded active principle (Rao et al., 2018; Zhuo et al., 2020; Ofridam et al., 2021). However, if the glass temperature of the hydrophobic sequence is too high, the core of the systems is glassy, thus freezing the structure and leading to the so-called “frozen-in” micelles, which can be assimilated to solid particles.

The intense research of the last four decades has allowed the development of extremely diverse polymer-drug systems, classified according to several criteria. One of them takes into account the release mechanism and kinetics of the active principle contained. We can thus distinguish three important categories, each of them being divided into other subcategories: (i) diffusion-controlled systems; (ii) erosion-controlled systems; (iii) osmosis-controlled systems. To these three main types, polymer systems are added to which the release of the active ingredient is controlled by ion exchange, or polymer-drug conjugates to which the release of the active ingredient is determined by the kinetics of the hydrolysis of chemical bonds between it and the macromolecular support (Shaik et al., 2012; Vilos & Velasquez, 2012).

Interactions taking place between the structural units of any polymer-drug system imply mutual constraints at different scale resolutions, nonlinearity being one of the most fundamental properties of any complex system dynamics. The universality of the dynamics laws for any polymer-drug system becomes natural. Some of the usual theoretical models describing these systems dynamics are based on the hypothesis that the variables characterizing the polymer-drug system dynamics are differentiable, which can be otherwise unjustified. From such a perspective, validations of these models must be seen as sequential and applicable on restricted domains, for which integrability and differentiability are respected.

Formal kinetic models have been developed for each of these types of systems. Widely used in this context is the Ritger-Peppas kinetic model of (Ritger & Peppas, 1987), based essentially on Fick’s law:

$$M_{t}/M_{0}=k\times t^{n}$$

where $M_{t}$ is the amount of drug released at time t of the process, $M_{0}$ is the total amount of drug encapsulated in the system, k is the rate of the release process and n is an exponential factor with a value between 0 and 1. Depending on the value of n, predictions about the release mechanism are possible.

Since nonlinearity is implying that, in the description of polymer-drug system dynamics, non-differentiable behaviors are predominant, it is necessary to explicitly introduce the scale resolution in equations defining the variables governing these dynamics. This leads to the fact that any variables used in the description of any complex system have a dual dependence, both on the space-time coordinates and the scale resolution. In this new perspective, instead of using variables defined by non-differentiable functions, approximations of these polymer-drug system functions will be used at various scale resolutions. Therefore, all variables used to define the afore-mentioned dynamics will work as a limit of families of functions, being non-differentiable at a null scale resolution, and differentiable for non-null scale resolution. Thus, suitable geometrical structures and a class of models for which the motion laws are integrated with scale laws must be developed. These geometrical structures are constructed on the notion of multifractality, the equivalent theoretical modes being based on the Scale Relativity Theory (SRT), either with fractal dimension *D*_F_ = 2 or in an arbitrary and constant fractal dimension (The Multifractal Theory of Motion). In the case of such (non-differentiable) models, the polymer-drug system’s structural units dynamics can be described by continuous but non-differentiable movement curves (multifractal motion curves). The obtained curves exhibit self-similarity as their main property in any of their points, which translates into holographic-type behaviors (every part reflects the global system). Such a complex approach suggests that only a holographic implementation can provide a complete description of the polymer-drug system dynamics (Mazilu et al., 2021; Agop & Merches, 2019).

In the framework of SRT (Nottale, 2011) and The Multifractal Theory of Motion (Mercheș & Agop, 2016; Agop & Paun, 2017), if we assimilate any polymer-drug system with a fractal-type mathematical object, various non-linear behaviors through a fractal hydrodynamic-type description as well as through a fractal Schrödinger-type description can be established (Mercheș & Agop, 2016; Agop & Paun, 2017). Thus, the fractal hydrodynamic-type description implies holographic implementations for dynamics through velocity fields at non-differentiable scale resolution, via fractal soliton, fractal soliton-kink and fractal minimal vortex. In this context, various operational procedures can become functional. Several of these procedures are of particular notice: the fractal cubics with fractal SL(2R) group invariance through in-phase coherence of the structural units dynamics of any polymer-drug system; the fractal SL(2R) groups through dynamics synchronization along the polymer-drug systems structural units; the fractal Riemann manifolds induced by fractal cubics and embedded with a Poincaré metric through apolar transport of cubics (parallel transport of direction, in a Levi Civita sense); the harmonic mapping from the usual space to the hyperbolic one. These procedures become operational so that one can obtain several possible scenarios toward chaos (fractal periodic doubling scenario), but without fully transitioning into chaos (non-manifest chaos).

Our research team has already studied the micellization and the preparation of a curcumin-loaded micellar system based on “frozen-in” poly(2-vinylpyridine)-b-poly(ethylene oxide) (P2VP-b-PEO) pH-sensitive diblock copolymers (Iurciuc-Tincu et al., 2020). From these studies, it appeared that the micellar disaggregation occurs at a pH value of 4.5, which is the pKa of the P2VP sequence. Also, at a pH value smaller than the pKa, the curcumin release is more rapid due to the disintegration of the micelles than at pH 7.4. However, the literature is poor in investigating the drug release kinetics of such structures.

In this work, we analyzed, from a multifractal perspective, the nonlinear dynamics of complex systems, generalizing the results from (Ailincai et al., 2021; Iftime et al., 2020). In such context, by exploring a hidden symmetry in the form of synchronization groups of polymer-drug system entities, we were led to the generation of a Riemann manifold with hyperbolic type metric via parallel direction of transport. Then, the polymer-drug systems nonstationary dynamics were highlighted through harmonic mapping from the usual space to the hyperbolic one.

## 2. Experimental section

### 2.1. Synthesis procedure of copolymer samples

Poly(2-vinylpyridine)-b-poly(ethylene oxide), P2VP-b-PEO, diblock copolymers were synthesized by living anionic polymerization in THF in the presence of phenylisopropyl potassium as initiator (Atanase & Riess, 2013). For decreasing the reactivity of the initiator and stopping the transfer reactions, a unit of 1,1-diphenylethylene is recommended. First the 2-vinylpyridine is polymerized at −75 °C for 2.5 h. The ethylene oxide is added and the temperature is increased to 20 °C. The copolymer was recovered by precipitation in heptane, followed by drying in vacuum.

The molecular characteristics of the studied copolymers are presented in Table 1.

**Table 1.** Block copolymer molecular characteristics.

Samples	**M_n_(P2VP) (g/mol)** ^a^	**M_n_(PEO) (g/mol)** ^b^	M_n_total (g/mol)	M_w_/M_n_	**wt% P2VP** ^c^
**A**	5800	12500	18300	1.11	31.6
**B**	9500	17500	27000	1.09	35.0
**C**	28800	61900	90700	1.29	31.7

a *by SEC data*.

b *^,^*^c^*by ^1^H NMR*.

### 2.2. Preparation and characterization of drug-loaded micelles

The drug-loaded micelles were prepared by dialysis method using a common solvent. In a typical experiment, 250 mg of each type of P2VP-b-PEO block copolymer sample and 25 mg of Curcumin were dissolved in 50 ml of dimethylsulfoxide solution (DMSO) at room temperature, under stirring and in dark. After complete dissolution of the powder, the solution was dialyzed, in the dark, for 24 h against ultrapure water using dialysis cellulose membranes (molecular weight: 12 kDa, manufacturer, Sigma). After this period, the micellar solution from the dialysis membrane was collected, frozen and then lyophilized to obtain a dry powder which was stored at −4 °C prior to use.

**Figure 1.**
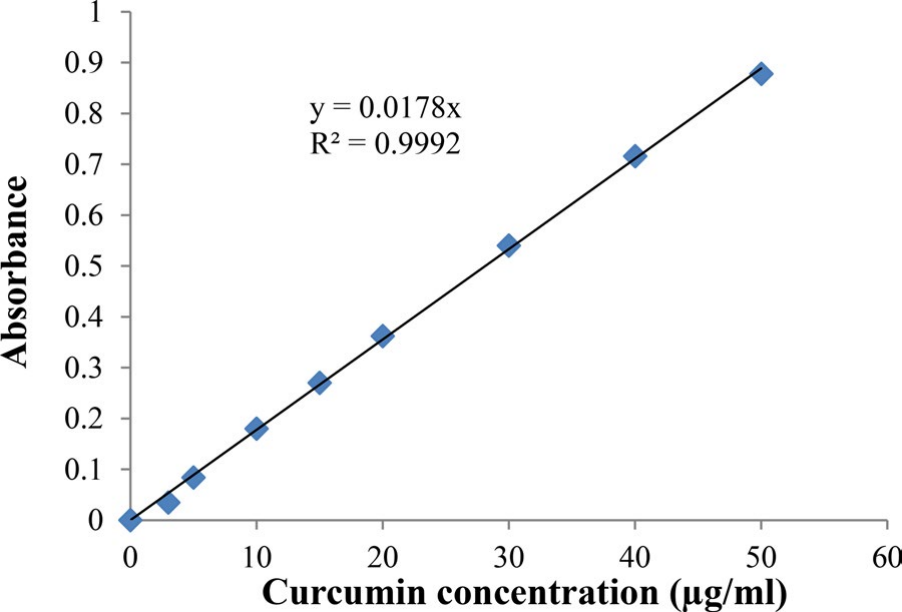
Calibration curve of Curcumin in DMSO.

To determine the loading efficiency of Curcumin, a calibration curve was plotted in DMSO, using different Curcumin concentrations and their absorbance was recorded on a Nanodrop spectrophotometer at a wavelength of 435 nm. The calibration curve equation was y = 0.0178x, R^2^ = 0.9992, as illustrated in Figure 1.

The amount of Curcumin in 10 mg micelles was extracted into DMSO using the following protocol: a known quantity of drug-loaded micelles was dispersed in 5 ml DMSO and then added to dialysis membranes. The samples thus prepared were added in a known volume of DMSO (20 ml) and kept under stirring in a water bath in Erlenmeyer flasks at 37 °C in the dark. The absorbance was read after 24 h, when its value remained constant, Curcumin being completely extracted in DMSO from micelles. Based on the calibration curve, both drug loading (DLE) and drug encapsulation (DEE) efficiencies were determined using the following equations:

(1) $$\mathrm{DLE}(\%)=\frac{\mathrm{Amount}of\mathrm{drug}in\mathrm{micelles}}{\mathrm{Amount}of\mathrm{added}\mathrm{polymer}+\mathrm{drug}}x100$$

(2) $$\mathrm{DEE}(\%)=\frac{\mathrm{Amount}\text{ of }\mathrm{drug}\text{ in }\mathrm{micelles}}{\mathrm{Amount}\text{ of }\mathrm{added}\mathrm{drug}}x100$$

### 2.3. Evaluation of drug release kinetics

The kinetics of curcumin release from micelles was studied in three different pH environments: in phosphate buffer solution (PBS) 0.1 M at pH = 7.4 (specific for blood and colon fluids), in PBS 0.1 M at pH = 6.8 which mimics the fluid intestinal, solution at pH = 2 (solution prepared from 10 mM NaCl and HCl − 0.1 N) which is specific to the pH of the gastric environment.

In a typical experiment, 10 mg of the drug-loaded micelles with were dispersed in 5 ml of buffer solution of different pH and then added to a dialysis membrane. The suspension thus prepared was added to an Erlenmeyer flask and immersed in 20 ml PBS at a given pH value, under stirring, at 37 °C in the dark. At a given time, samples were taken in order to determine spectrophotometrically the absorbance at the Curcumin-specific wavelength of 425 nm. As Curcumin is a hydrophobic substance and is very slightly soluble in aqueous media, 1% (w/w) Tween 20 was added to release medium.

To avoid the degrading action of light on Curcumin, all experiments were conducted in the dark (dialysis, kinetic study of the release process). The vials in which the experiments were performed were covered with an aluminum foil throughout.

### 2.4. Theoretical design

Our theoretical model is based on the Scale Relativity Theory (Nottale, 2011; Mercheș & Agop, 2016; Agop & Paun, 2017). This theory has been successfully used for describing the dynamics of complex systems, and, in particular, for modeling release dynamics (Ailincai et al., 2021; Iftime et al., 2020). The main assumption of this theory is that any polymer-drug system, as a complex system, can be assimilated with a fractal/multifractal mathematical object (Mandelbrot, 1982; Jackson, 1993; Cristescu, 2008). The functionality of such a hypothesis implies, based on the Scale Relativity Theory, employing, in the description of polymer-drug dynamics, continuous and non-differential curves (fractal/multifractal curves). Then, two scenarios for describing polymer-drug dynamics become compatible:

- release dynamics in the Schrödinger multifractal scenario (multifractal Schrödinger equation) (Peptu et al., 2021):
(3) $$\lambda^{2}(dt{)}^{[\frac{4}{f(\alpha )}]-2}\partial^{l}\partial_{l}\Psi +i\lambda (dt{)}^{[\frac{2}{f(\alpha )}]-1}\partial_{t}\Psi =0$$
- release dynamics in the Madelung multifractal scenario (multifractal hydrodynamic equation) (Peptu et al., 2021):
(4) $$\partial_{t}V_{D}^{i}+V_{D}^{l}\partial_{l}V_{D}^{i}=-\partial^{i}Q$$
(5) $$\partial_{t}\rho +\partial_{l}(\rho V_{D}^{l})=0$$

where Q denotes the multifractal specific potential:

(6) $$Q=-2\lambda^{2}{\left(dt\right)}^{[\frac{4}{f(\alpha )}]-2}\frac{\partial^{l}\partial_{l}\sqrt{\rho }}{\sqrt{\rho }}=-V_{F}^{i}V_{F}^{i}-\frac{1}{2}{\left(dt\right)}^{[\frac{2}{f(\alpha )}]-1}\partial_{l}V_{F}^{l}$$

Equation (4) corresponds to the multifractal momentum conservation law, while equation (5) corresponds to the multifractal states density conservation law.

In relations (3) – (6),

(7) $$\Psi =\sqrt{\rho }e^{is}$$

(8) $$V_{D}^{i}=2\lambda {\left(dt\right)}^{[\frac{2}{f(\alpha )}]-1}\partial^{i}s$$

(9) $$V_{F}^{i}=\lambda {\left(dt\right)}^{[\frac{2}{f(\alpha )}]-1}\partial^{i}\ln\rho$$

(10) $$\partial_{t}=\frac{\partial }{\partial t},\partial_{l}=\frac{\partial }{\partial x^{l}},\partial_{l}\partial_{k}=\frac{\partial }{\partial x^{l}}\frac{\partial }{\partial x^{k}},i=\sqrt{-1},l,k=1,2,3$$

and the quantities from (3) – (10) have the following meanings:

- $x^{l}$ is the multifractal spatial coordinate;
- t is the non-multifractal time with the role of an affine parameter of the motion curves;
- dt is the scale resolution;
- Ψ is the state function of amplitude $\sqrt{\rho }$ and phase *s*;
- $V_{D}^{l}$ is the differentiable velocity independent of scale resolution;
- $V_{F}^{l}$ is the non-differentiable velocity dependent of scale resolution;
- f(α) is the singularity spectrum of order α;
- α is the singularity index and it is a function of fractal dimension $D_{f}$;
- λ is a coefficient associated to the multifractal/non-multifractal scale transition

In fact, the two scenarios describing the dynamics of drug release are not mutually excluding, but on the contrary, they are complementary.

### 2.5. Drug release mechanisms through synchronization modes

Since the multifractal specific potential *Q* can be put into relation with the multifractal tensor

(11) $$\overset{\wedge }{\tau^{il}}=2\lambda^{2}(dt{)}^{[\frac{4}{f(\alpha )}]-2}\rho \partial^{i}\partial^{l}\ln\rho$$

in the form

(12) $$\rho \partial^{i}Q=\partial_{l}\overset{\wedge }{\tau^{il}}$$

it is natural to admit that tensor (11) becomes fundamental in drug release processes. Then, its characteristic equations are given by the cubics:

(13) $$a_{0}X^{3}+3a_{1}X^{2}+3a_{2}X+a_{3}=0,a_{0},a_{1}a_{2}a_{3}\in R$$

If (13) has real roots (Agop & Merches, 2019; Mazilu & Agop, 2014):

(14) $$X_{1}=\frac{h+\bar{h}k}{1+k},X_{2}=\frac{h+\varepsilon \bar{h}k}{1+\varepsilon k},X_{3}=\frac{h+\varepsilon^{2}\bar{h}k}{1+\varepsilon^{2}k}$$

with h, $\bar{h}$ the roots of Hessian and $\varepsilon \equiv (-1+i\sqrt{3})/2$ the cubic root of unity $\left(i=\sqrt{-1}\right)$, the values of variables h, $\bar{h}$ and k can be “scanned” by a simple transitive group with real parameters (Agop & Merches, 2019; Mazilu & Agop, 2014). This group can be revealed through Riemann-type spaces associated with the previous cubic. The basis of this approach is the fact that the simply transitive group with real parameters (Agop & Merches, 2019; Mazilu & Agop, 2014):

(15) $$X_{l}↔\frac{aX_{l}+b}{cX_{l}+d},l=1,2,3a,b,c,d\in R$$

where $X_{l}$ are the roots of the cubic (13), induces the simply transitive group in the variables h, $\bar{h}$ and k, whose actions are:

(16) $$h↔\frac{ah+b}{ch+d},\bar{h}↔\frac{a\bar{h}+b}{c\bar{h}+d},k↔\frac{c\bar{h}+d}{ch+d}k$$

The structure of this group is of SL(2R) - type

(17) $$[B^{1},B^{2}]=B^{1},[B^{2},B^{3}]=B^{3},[B^{3},B^{1}]={-2B}^{2}$$

where $B^{l}$ are the infinitezimal generators of the group:

(18) $$\begin{array}{c} B^{1}=\frac{\partial }{\partial h}+\frac{\partial }{\partial \bar{h}}\\ B^{2}=h\frac{\partial }{\partial h}+\bar{h}\frac{\partial }{\partial \bar{h}}\\ B^{3}=h^{2}\frac{\partial }{\partial h}+{\bar{h}}^{2}\frac{\partial }{\partial \bar{h}}+(h-\bar{h})k\frac{\partial }{\partial k} \end{array}$$

This group admits the differential 1-forms:

(19) $$\begin{array}{c} \omega^{1}=\frac{dh}{(h-\bar{h})k}\\ \omega^{2}=-i(\frac{dk}{k}-\frac{dh+d\bar{h}}{h-\bar{h}})\\ \omega^{3}=-\frac{kd\bar{h}}{h-\bar{h}} \end{array}$$

and the differential 2-forms (the metric):

(20) $$ds^{2}={\left(\frac{dk}{k}-\frac{dh+d\bar{h}}{h-\bar{h}}\right)}^{2}-4\frac{\mathrm{dhd}\bar{h}}{{\left(h-\bar{h}\right)}^{2}}$$

In real terms

(21) $$h=u+iv,\bar{h}=u+iv,k=e^{i\theta }$$

and for

(22) $$\begin{array}{c} \Omega^{1}=\omega^{2}=d\theta +\frac{du}{v}\\ \Omega^{2}=\text{cos}\theta \frac{du}{v}+\text{sin}\theta \frac{dv}{v}\\ \Omega^{3}=-\text{sin}\theta \frac{du}{v}+\text{cos}\theta \frac{dv}{v}, \end{array}$$

the connection with the Poincaré representation of the Lobachevsky plane can be highlighted in the form:

(23) $$ds^{2}=-{\left(\Omega^{1}\right)}^{2}+{\left(\Omega^{2}\right)}^{2}+{\left(\Omega^{3}\right)}^{2}=-{\left(d\theta +\frac{du}{v}\right)}^{2}+\frac{du^{2}+dv^{2}}{v^{2}}$$

This metric reduces to that of Poincaré for $\Omega^{1}\equiv 0$, i.e.,

(24) $$\frac{dk}{k}-\frac{dh+d\bar{h}}{h-\bar{h}}=0↔d\theta =-\frac{du}{v}$$

Then, we can define the variable θ as the „angle of parallelism” of the hyperbolic planes (the connection). In such a conjecture, it is noted that, if the cubic is assumed to have distinct roots, the condition (24) is satisfied, if, and only if, the differential forms $\Omega^{1}$ is null.

Therefore, for the metric (23) with restriction (24), the relation becomes:

(25) $$ds^{2}=\frac{\mathrm{dhd}\bar{h}}{{\left(h-\bar{h}\right)}^{2}}=\frac{du^{2}+dv^{2}}{v^{2}}$$

The parallel transport of the hyperbolic plane actually represents the apolar transport of the cubics .

Therefore, the group (16) can be assimilated with a “synchronization” group between the different structural units (entities) of the polymer-drug system. In this process, the amplitude of each of the entity of any polymer-drug system participates, in the sense that they are correlated. Moreover, the phases of any entity of the polymer-drug system are also correlated. The usual synchronization, manifested through the phase shift of the polymer-drug system entities, is, in this case, only a very particular case.

In the following, non-stationary dynamics in complex systems through harmonic mappings will be generated. Indeed, let it be assumed that the complex system dynamics are described by the variables $\left(Y^{j}\right)$, for which the following multifractal metric was discovered:

(26) $$h_{ij}dY^{i}dY^{j}$$

in an ambient space of multifractal metric:

(27) $$\gamma_{\alpha \beta }dX^{\alpha }dX^{\beta }$$

In this situation, the field equations of the complex system dynamics are derived from a variational principle, connected to the multifractal Lagrangian:

(28) $$L=\gamma^{\alpha \beta }h_{ij}\frac{dY^{i}dY^{j}}{\partial X^{\alpha }\partial X^{\beta }}$$

In the current case, (26) is given by (25) with the constraint (24), the field multifractal variables being h and $\bar{h}$ or, equivalently, the real and imaginary part of *h*. Therefore, if the variational principle:

(29) $$\delta \int L\sqrt{\gamma }d^{3}x$$

is accepted as a starting point where $\gamma =|\gamma_{\alpha \beta }|$, the main purpose of the polymer-drug system dynamics research would be to produce fractal/multifractal metrics of the multifractal Lobachevski plane (or relate to them). In such a context, the multifractal Euler equations corresponding to the variational principle (29) are:

(30) $$\begin{array}{c} (h-\bar{h})\nabla (\nabla h)=2{\left(\nabla h\right)}^{2}\\ (h-\bar{h})\nabla (\nabla \bar{h})=2{\left(\nabla \bar{h}\right)}^{2} \end{array}$$

which admits the solution:

(31) h=cosh⁡(Φ2)−sinh⁡(Φ2)e−iαcosh⁡(Φ2)+sinh⁡(Φ2)e−iα,α∈R

with α real and arbitrary, as long as (Φ2) is the solution of a Laplace-type equation for the free space, such that ∇2(Φ2)=0. For a choice of the form α=2Ωt, in which case a temporal dependency was introduced in the complex system dynamics, (31) becomes:

(32) $$h=\frac{i[e^{2\Phi }\;\text{sin}\;\left(2\Omega t\right)-\;\text{sin}\;(2\Omega t)-2ie^{\Phi }]}{e^{2\Phi }[\;\text{cos}\;\left(2\Omega t\right)+1]-\;\text{cos}\;\left(2\Omega t\right)+1}$$

**Figure 2.**
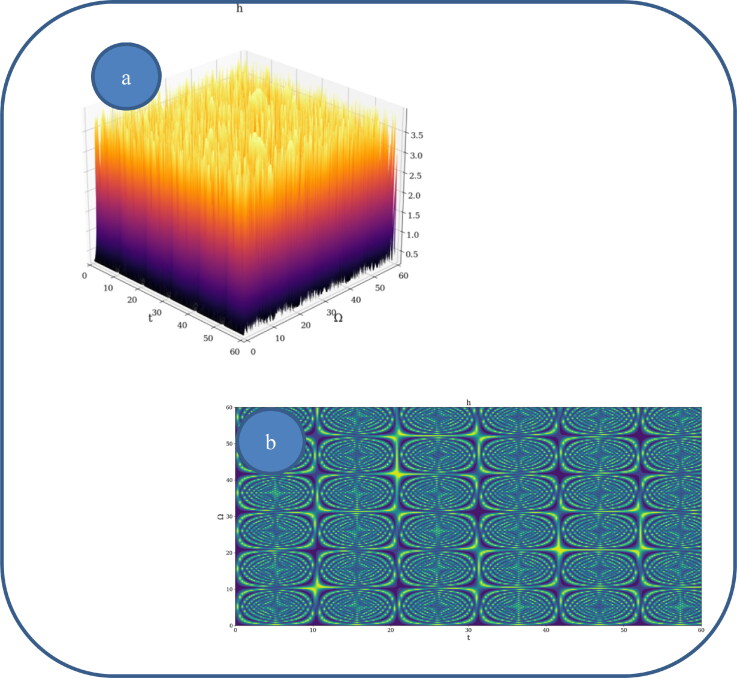
**a, b:** Drug delivery modes at global scale resolution plot of h(Ω,t) with Φ=2.35: 3 D representation (a) and 2 D representation (b).

**Figure 3.**
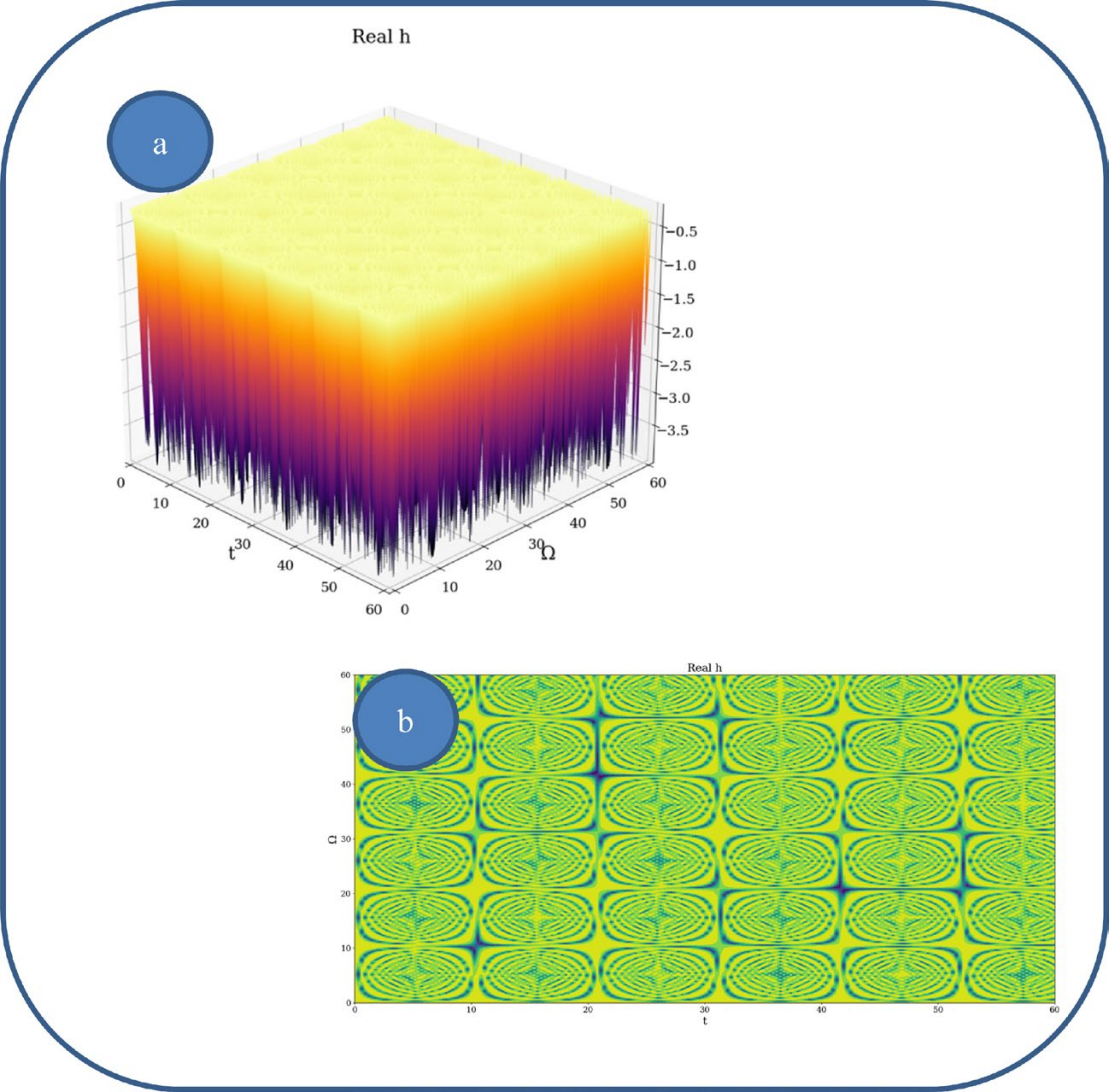
**a, b**: Drug delivery modes at differentiable scale resolution plot of Re[h(Ω,t)] with Φ=2.35: 3 D representation (a) and 2 D representation (b).

**Figure 4.**
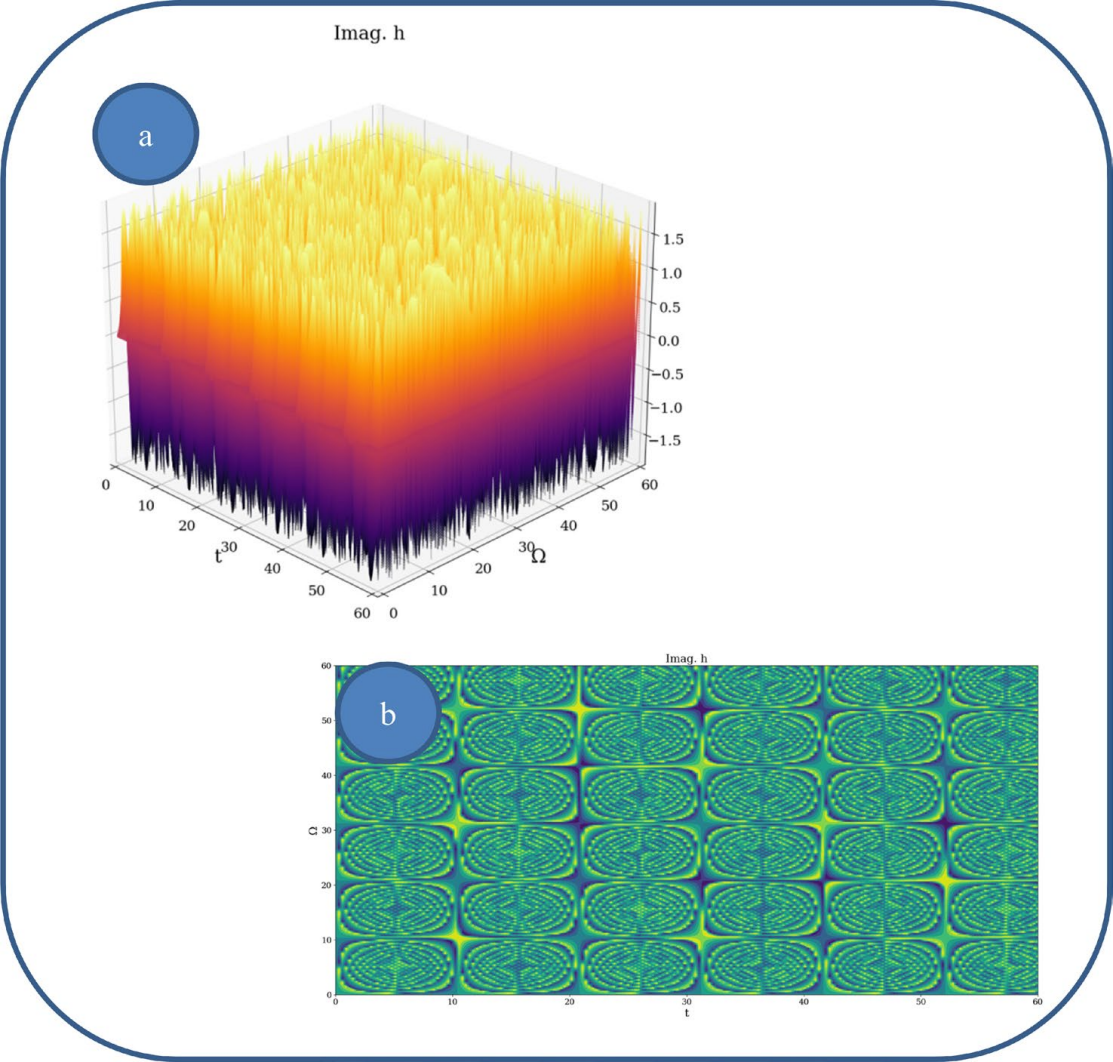
**a, b**: Drug delivery modes at non-differentiable scale resolution plot of Im[h(Ω,t)] with Φ=2.35: 3 D representation (a) and 2 D representation (b).

In Figures 2 and 3, various nonlinear drug delivery modes at different scale resolutions in dimensionless coordinates are presented: (i) for global scale resolution (Figure 2a and b); (ii) for differentiable scale resolution (Figure 3a and b); (iii) for non-differentiable scale resolution (Figures 4a and b). Let it be noted that, whatever the scale resolution, the drug release dynamics prove themselves to be reducible to self-structuring patterns. The structures are present in pairs of two large patterns that communicate in an intermittent way. In the 0-20 range for *Ω* and *t* the resulted structures are communicating with each other via a channel created along the symmetry axis for t ∼ 10. This channel is also seen for different (*Ω;t*) coordinates which is interpreted as an intermittency in the structure bonding.

**Figure 5.**
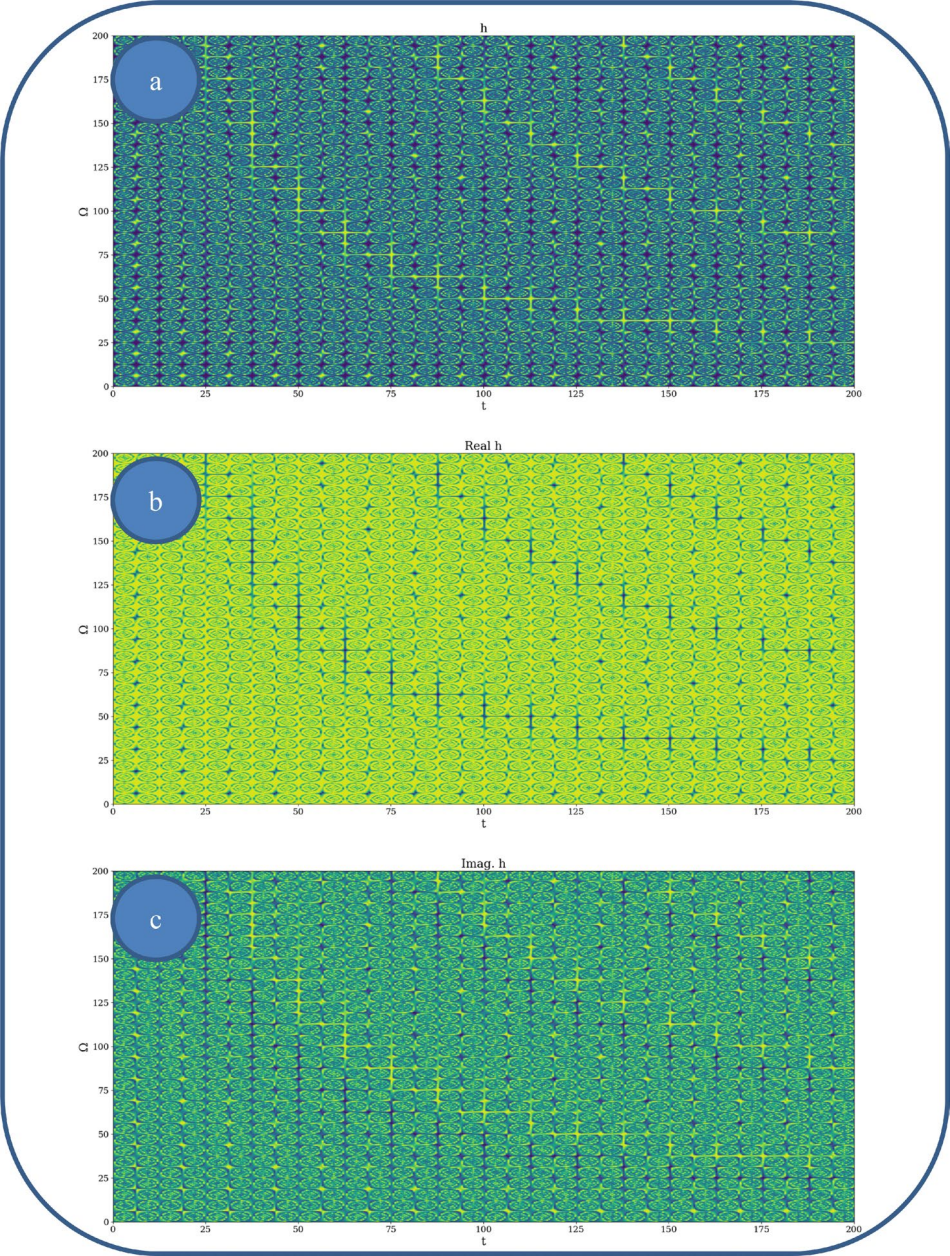
**a–c**: 2 D polymer-drug dynamics at global scale resolution plot of: (a) h;(b)Re(h);(c) Im (h) *for* (Ω=0−200,t=0−200)and Φ =2.35.

**Figure 6.**
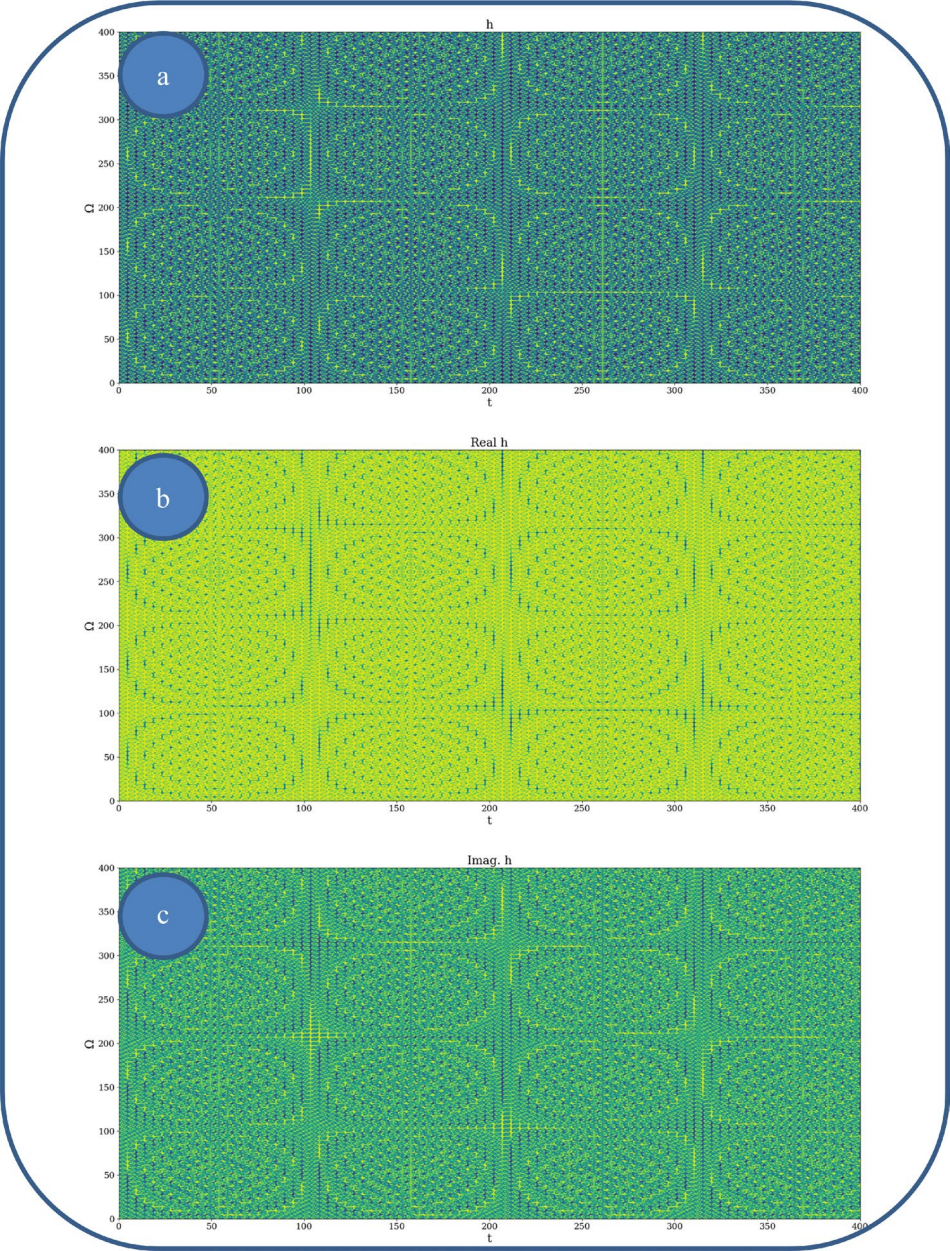
**a–c**: 2 D polymer-drug dynamics at global scale resolution plot of h,Re(h)and Im (h)(Ω=0−400,t=0−400); Φ=2.35.

**Figure 7.**
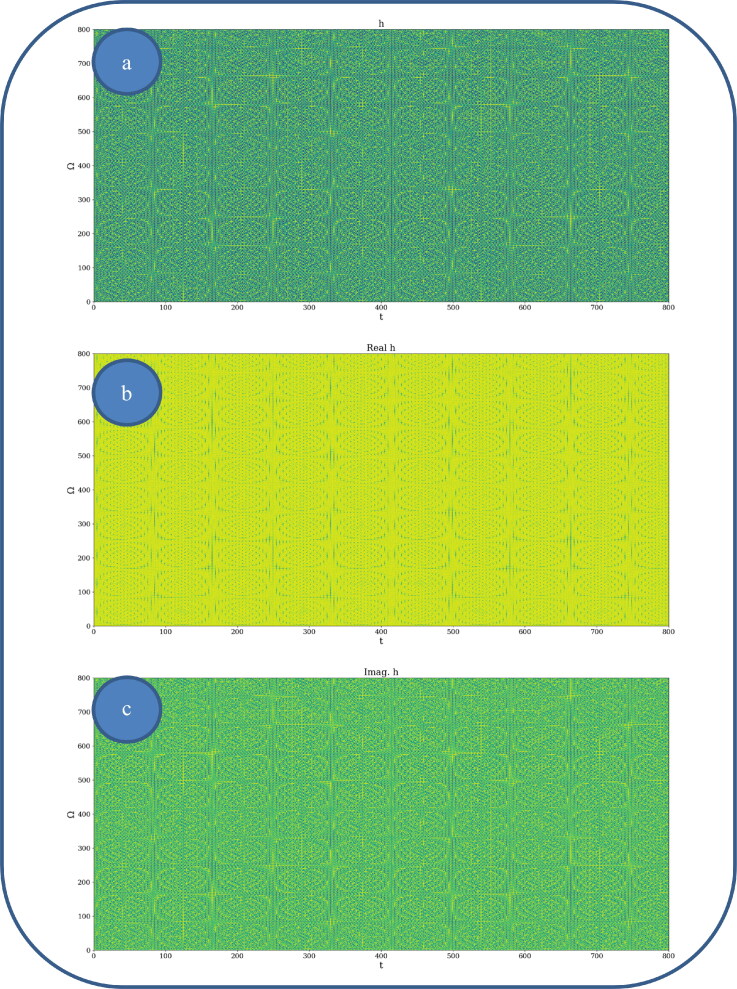
**a–c**: 2 D polymer-drug dynamics at global scale resolution plot of h,Re(h)and Im (h)(Ω=0−800,t=0−800); Φ=2.35.

We present in Figures 5–7, by plotting h in dimensionless parameters, certain temporal self-similar properties of the polymer-drug dynamics. It can be observed that the multifractal structures are contained into similar multifractal structures at much higher scales. Moreover, since the structure’s communication channel has an exponential decrease in the (*Ω;t*) plane, dissipation processes (Jackson, 1993; Cristescu, 2008) occurring during drug release are present. The model manages to express the dissipation of the drug through the reduction of the channel amplitude on the *Ω* axis as the time variable is increased.

From these previous figures, one can also notice channel-type patterns through self-structuring of the polymer-drug system entities.

## 3. Results

### 3.1. Modeling of the drug release mechanism

The obtained results concerning the drug loading and encapsulation efficiencies are given in Table 2.

**Table 2.** DLE and DEE values for all copolymer samples.

Sample	Curcumin/copolymer mg/g	DLE (%)	DEE (%)
A	65.30	6.53	71.83
B	63.95	6.40	70.35
C	51.26	5.13	56.39

As can be observed from this table, the DLE and DEE are related to the molecular characteristic of the copolymers. Sample A, with the smallest molecular weight, has the highest DLE and DEE values. On the contrary, sample C has the lowest efficiencies having the highest molecular weight.

**Figure 8.**
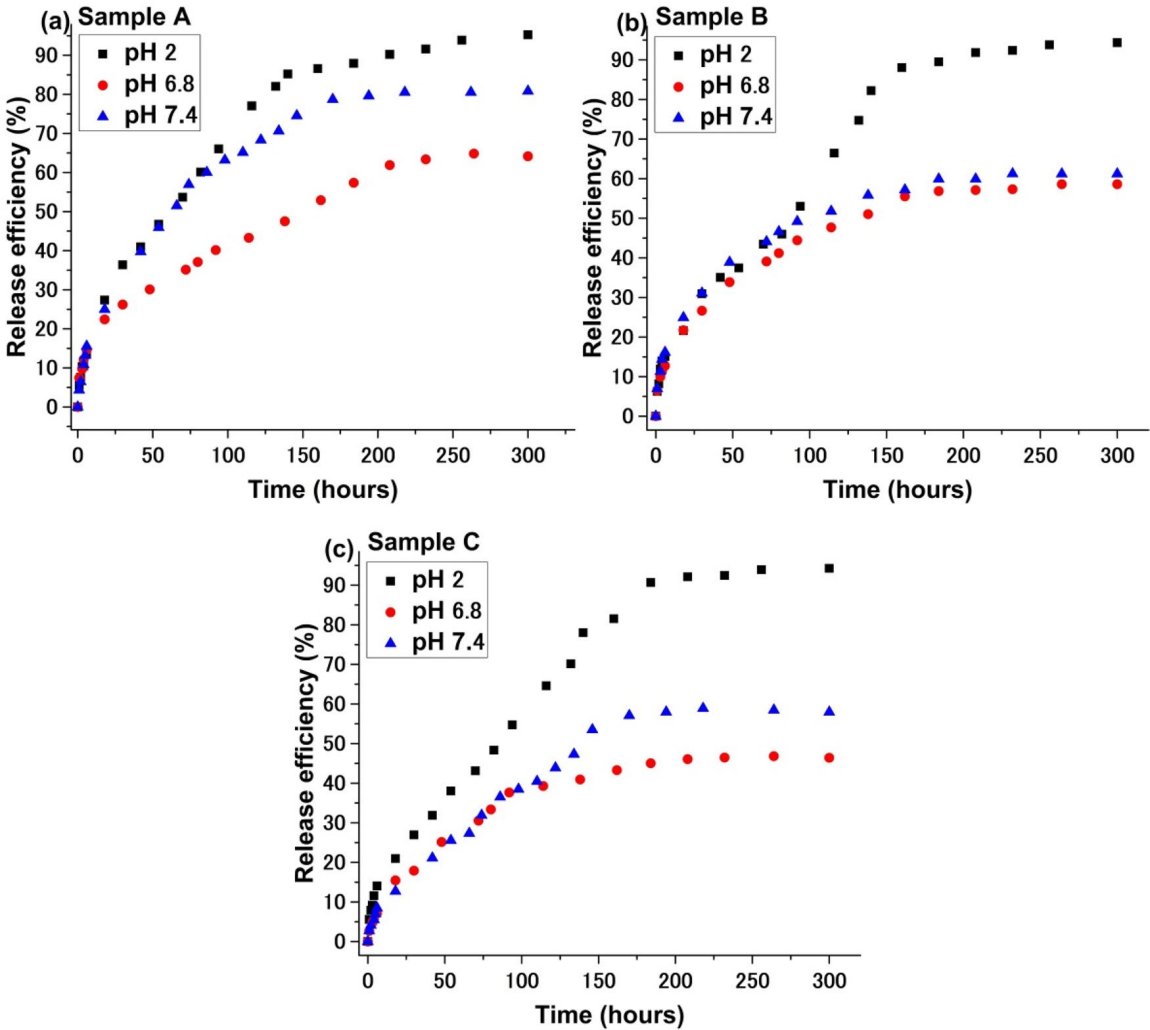
Drug release curves for all three samples.

**Figure 9.**
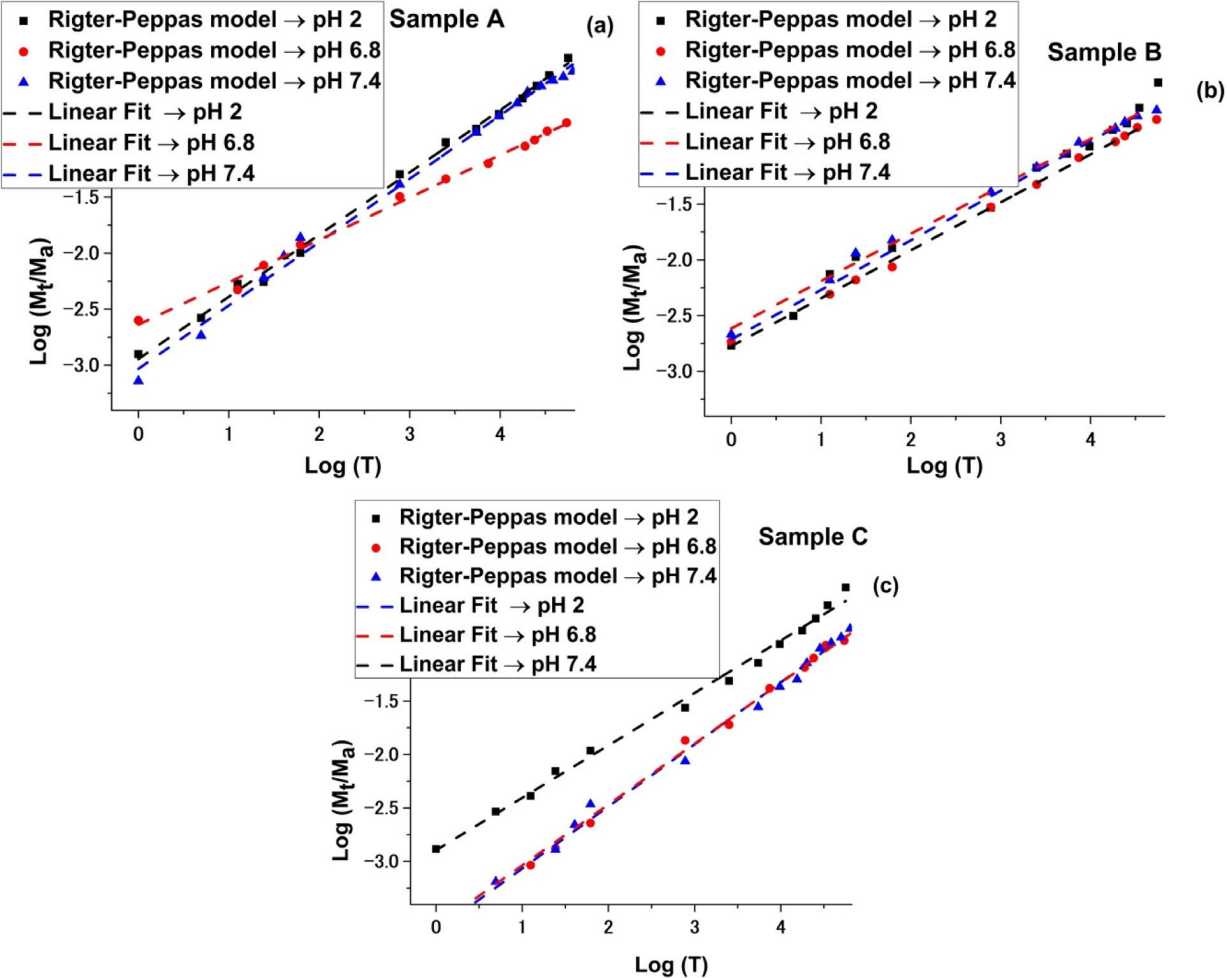
Ritger-Peppas kinetic mode implemented for the drug release curves for all three samples.

In Figures 8 and 9, the drug release kinetics as a function of pH for all three samples are illustrated.

From Figure 8, it appears that the Curcumin release kinetics is strongly influenced by the pH, as expected. At pH 2, the P2VP sequence is protonated and therefore a demicelization process occurs leading to the destruction of the micelles and thus to the almost complete release of loaded drug. At other pH values, the micelles are “frozen-in” and the Curcumin release is controlled over time.

Information on the mechanism of transport and release of the active substance from the micelles was obtained using the Ritger-Peppas kinetic model ($\frac{M_{t}}{M_{\infty }}=k1\times t^{n}$) and the resulted plots are represented in Figure 9. The resulted data is synthesized in Table 3.

**Table 3.** Centralization of the results from the Ritger-Peppas kinetic model implementation.

*pH*	*Sample*	*Ef (%)*	*Time (h)*	*n*	*R^2^*
2	A	95.2	300	0.548	0.9868
	B	94.3	300	0.445	0.9902
	C	94.1	300	0.527	0.9902
6.8	A	63.0	264	0.417	0.9870
	B	58.2	264	0.428	0.9989
	C	46.8	264	0.519	0.9879
7.4	A	80.8	218	0.544	0.9922
	B	61.6	264	0.410	0.9954
	C	58.8	218	0.566	0.9855

The strong acidic environment strongly destabilizes the micelles, regardless of the molecular and compositional characteristics of the amphiphilic copolymer used. The effectiveness of releasing the active ingredient is maximum after around 5 hours of experiment. An environment close to the neutral pH value is reducing the intensity of the release process, which becomes the lowest in the case of micelles formed by the copolymer with the highest molecular weight. A possible interpretation must be related to the larger size of the micelle core, on one hand, which reduces the diffusion intensity of the active principle, and perhaps to the length of the copolymer PEO sequences that stabilizes the micelle: a longer length of these sequences will also slow the release of Curcumin. Interesting is the behavior at the physiological value of pH. Regardless of the value of this characteristic, the efficiency of the release process is superior to the weakly acidic environment (pH = 6.8) due to a better solubilization of Curcumin. The value of the exponential coefficient, n, is around 0.5, generally suggesting a diffusion process, slightly disturbed in some cases.

### 3.2. The synergy of the two models describing the release dynamics through the scale relativity theory

As we have previously shown, the two fractal/multifractal scenarios of the dynamics of polymer-drug systems are not mutually exclusive, but, on the contrary, are complementary. Thus, if the hydrodynamic scenario allows the “decoding” of some drug release mechanisms, then the Schrodinger scenario can be employed for the “plotting” of the drug release curves. For a better understanding of our argumentation, let us remind the hypotheses from (Peptu et al., 2021):

- the dynamics of any complex system, independent of the two scale resolutions (differentiable and non-differentiable scale resolutions), are one-dimensional dynamics;
- the synchronization of the dynamics of any complex system at the two scale resolutions is achieved by “compensating” the velocity fields $V_{D}^{l}$ and $V_{F}^{l}$ given by the restriction $V_{D}^{l}=-V_{F}^{l}$;
- the polymer network operates by means of a potential vector $A_{x}=\frac{1}{2}Bx,B=\mathrm{const}.$ (for details, see (Peptu et al., 2021)).

Taking the above hypotheses into consideration, the multifractal states density conservation law becomes a multifractal Fokker-Planck-type equation:

(33) $$\partial_{t}\rho +\partial_{x}(-\eta x\rho )+\partial_{xx}(-\frac{D}{2}\rho )[-\frac{D}{2}(dt{)}^{[\frac{2}{f(\alpha )}]-1}\rho ]=0$$

where $D=2\lambda (dt{)}^{[\frac{2}{f(\alpha )}]-1}$, $\eta =\frac{1}{2}gB$, and *g* a coupling constant. The meanings of these quantities are given in (Peptu et al., 2021).

The solution of equation (33) has the expression:

(34) $$\rho (x,t)={\left(\frac{1}{2\pi (\frac{D}{\eta })[1-\;\text{exp}\;(-2\eta t)]}\right)}^{\frac{1}{2}}\;\text{exp}\;\left[-\frac{{\left[x-x_{0}\;\text{exp}\;(-\eta t)\right]}^{2}}{2(\frac{D}{\eta })[1-\;\text{exp}\;(-2\eta t)]}\right],x_{0}=\mathrm{const}.$$

signifying that the states density is a multifractal Gaussian whose average multifractal value decreases exponentially to zero and whose multifractal variance tends asymptotically toward (D/η) .

**Figure 10.**
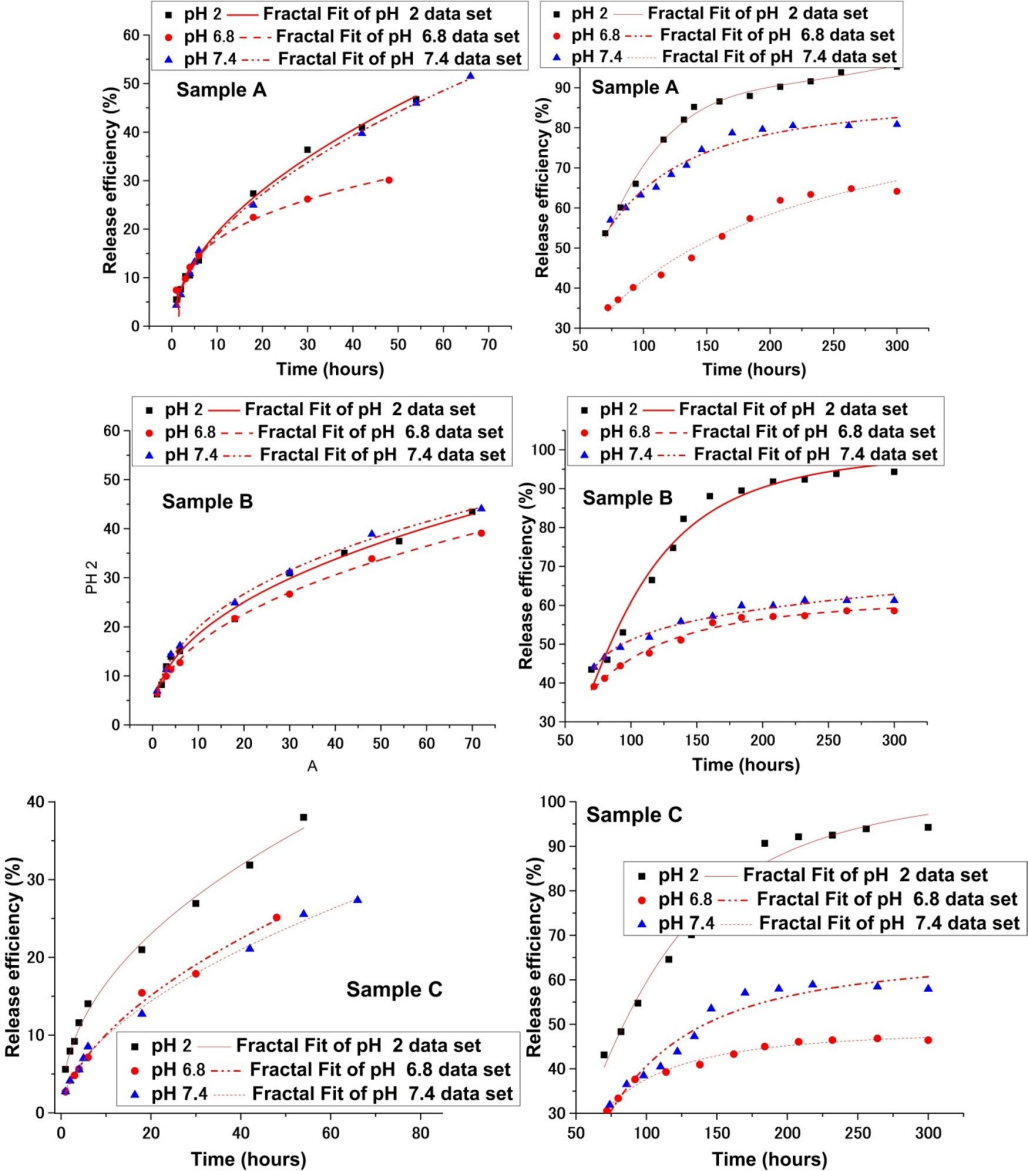
Fractal fit of the drug release curves at two different temporal scales < 50 h (left hand side of the figure) and >50 h (right hand side of the figure).

In Figure 10, we have fitted the experimental drug release date with our multifractal model. Based on the premise of our model the release dynamics can be seen here manifested at two temporal scale resolutions. The first one corresponds to about 30% of the overall release mass and can be attributed to release dynamics with on which there are small to none external restrictions. Translating this in the multifractal framework where the model was developed it means the geodesics defining the movement of the drug particles a not “broken” significantly and should be defined by lower fractalization degree. This is seen in Table 4 where the data on fractalization degrees is synthesized. We observe that fractalization degree at lower scale resolutions is with a factor of 3-4 lower than the ones computed at larger scale resolutions. This means that when implementing the “zoom in” we can find dynamics which are completely different from the ones dominating the large-scale dynamics. This is understandable as the simulations presented in Figure 4 show localized dynamics for fixed fractalization values. The large scale resolutions dynamics lead to derived values of the fractalization degree considerably higher. This is understandable as the values represent a cumulative characterization of the system. The values of the fractalization of the system are inverse proportional to the efficiency maximum computed. This is understandable as lower fractalization media are proper environments for fast dynamics.

**Table 4.** Fractalization degree computed for samples at two resolution scales.

	A		B		C	
pH	< 50 h	>50 h	< 50 h	>50 h	< 50 h	>50 h
**2**	5.2	10.4	2.4	10	1.3	9
**6.8**	3.1	3.8	2.3	4.8	1.4	6.2
**7.4**	5.1	8.6	2.04	4.53	1.1	6.5

## 4. Conclusions

The main results of this paper are the following:

- The release kinetics, as a function of pH, of a model active principle, i.e Curcumin, from nanomicelles obtained from amphiphilic pH-sensitive poly(2-vinylpyridine)-b-poly(ethylene oxide) (P2VP-b-PEO) tailor-made diblock copolymers was studied by using the Rietger-Peppas equation. The value of the exponential coefficient, n, is around 0.5, generally suggesting a diffusion process, slightly disturbed in some cases;
- By exploring a hidden symmetry in the form of synchronization groups of polymer-drug system entities, we were led to the generation of a Riemann manifold with hyperbolic type metric via parallel direction of transport. Then, the polymer-drug systems nonstationary dynamics were highlighted through harmonic mapping from the usual space to the hyperbolic one. In such context, various drug release modes, in the form of patterns and channels, become operational;
- The kinetic model developed based on fractal theory fits very well with the experimental data on the release of Curcumin from the amphiphilic copolymer micelles in which it was encapsulated, being a variant of the classical kinetic models based on the formal kinetics of the process. It also provides information on the mechanism of release of the biologically active principle from micellar nanocarriers, suggesting the structuring of the micelle according to the pH value, with the appearance of preferential channels through which the diffusion of the active principle occurs.
